# Older Asian Americans and Pacific Islanders with Activities of Daily Living (ADL) Limitations: Immigration and Other Factors Associated with Institutionalization 

**DOI:** 10.3390/ijerph9093264

**Published:** 2012-09-07

**Authors:** Esme Fuller-Thomson, Monica Chi

**Affiliations:** Factor Inwentash Faculty of Social Work, University of Toronto, 246 Bloor Street West, Toronto, ON M5S 1A1, Canada; Email: monica.chi@utoronto.ca

**Keywords:** older adults, Asian Americans, long-term care, immigration, activities of daily living, socio cultural factors, treatment barriers

## Abstract

This study determined the national prevalence and profile of Asian Americans with Activities of Daily Living (ADL) limitations and identified factors associated with institutionalization. Data were obtained from 2006 American Community Survey, which replaced the long-form of the US Census. The data are nationally representative of both institutionalized and community-dwelling older adults. Respondents were Vietnamese (n = 203), Korean (n = 131), Japanese (n = 193), Filipino (n = 309), Asian Indian (n = 169), Chinese (n = 404), Hawaiian/Pacific Islander (n = 54), and non-Hispanic whites (n = 55,040) aged 55 and over who all had ADL limitations. The prevalence of institutionalized among those with ADL limitations varies substantially from 4.7% of Asian Indians to 18.8% of Korean Americans with ADL limitations. Every AAPI group had a lower prevalence of institutionalization than disabled Non-Hispanic whites older adults (23.8%) (*p* < 0.001). After adjustment for socio-demographic characteristics, Asian Indians, Vietnamese, Japanese, Filipino, and Chinese had significantly lower odds of institutionalization than non-Hispanic whites (OR = 0.29, 0.31, 0.58, 0.51, 0.70, respectively). When the sample was restricted to AAPIs, the odds of institutionalization were higher among those who were older, unmarried, cognitively impaired and those who spoke English at home. This variation suggests that aggregating data across the AAPI groups obscures meaningful differences among these subpopulations and substantial inter-group differences may have important implications in the long-term care setting.

## 1. Introduction

Recently, increased research attention has been focused on the impact of immigration on health status [1,2,3]. However, most studies have focused on the health and health care utilization patterns of immigrants who are in young or middle adulthood. Less research has focused on the later-life health of immigrants, in general, and their use of nursing homes, in particular. Three quarters of Asian Americans are immigrants [4]. As of 2010, Asians comprised the largest percentage of incoming immigrants to the United States, surpassing Hispanics for the first time [5]. Thus, older Asian Americans are an important group to study.

Asian-American older adults are underrepresented in nursing homes [6,7,8]. Asian Americans face many barriers to nursing home utilization including language and food preferences and cultural norm that deter institutional care [9,10]. Although many family members do step forward to provide intensive caregiving for disabled elderly relatives, some Asian-Americans who must use nursing homes select it as the last resort and feel stigmatized that they do not have family members to provide care [8,11,12]. 

Although most Asians share these cultural norms and attitudes towards nursing homes, research indicates that Asian American subgroups substantially differ in morbidity, mortality and experience in health care utilization [13,14,15]. Asian groups also differ in culture, immigration history, socio-economic status, age distribution and English language skills [15,16]. These factors, in turn, influence health status [13], disability level [17], and nursing home utilization [18,19]. Unfortunately, due to sample size restrictions, Asians tend to be grouped together thereby obscuring important heterogeneity among these subpopulations [20]. Also, most studies that do look at ethno-specific Asian groups rely primarily on non-random samples [9,11,14].

There are many factors in addition to ethnicity which are associated with nursing home use. It is important to take these factors into account when the link between ethnicity and institutionalization is examined. Older age is strongly associated with higher levels of institutionalization [21]. One of the major predictors of nursing home use is the presence of Alzheimer’s disease or other cognitive disorders [21,22]. Other factors are associated with a lower likelihood of institutionalization including having a spouse to assist with meeting ADL needs. Immigrants without citizenship are not eligible for Medicaid nursing home coverage [11,14]. The cost of nursing homes may therefore be prohibitive to many non-citizens. Language spoken at home may also play a role. It reflects two factors: ease of accessing long-term care resources and level of acculturation. Individuals who speak English have a much easier time learning about and accessing long-term care. Non-anglophones may be less receptive to nursing home placement because they may fear social isolation if they are unable to communicate with staff and fellow residents. Further, level of acculturation may be associated with nursing home access. Use of traditional language at home is a common measure of acculturation [23,24]. Research suggests that Asians Americans who do not speak English at home are less acculturated [25]. Other studies indicate lower acculturation is associated with higher levels of filial piety, a set of Confucian values that emphasize the duties of the children, especially the eldest son, to care for the elderly parents [26]. Because Asian ethnicity is associated with higher filial norms [27], Asian ethnicity potentially decreases the use of long-term care through promotion of coresidence with adult children in the community. Based on the existing literature, we hypothesize that:

(1) The prevalence of institutionalization among those with ADL limitations will be significantly lower for each of the Asian groups in comparison to non-Hispanic whites and that significant difference will remain after accounting for differences in key risk factors.
(2) Among Asians, those who speak English at home will be more likely to be institutionalized
(3) There will be significant variation in the odds of institutionalization among the different Asian ethnicities, even after adjustments for key risk factors.
(4) Non-citizens will be less likely to be institutionalized than citizens.

This is the first nationally representative study of community dwelling and institutionalized Asians aged 55 years and over with Activities of Daily Living (ADL) limitations. We compared and contrasted Asian group differences and demographic characteristics, cognitive disability, language skills and citizenship. We examined the prevalence of and factors associated with institutionalization among Asian American groups. 

## 2. Methods

### 2.1. Data Source

Over three million Americans were interviewed by the U.S. Census Bureau in the 2006 American Community Survey (ACS) providing nationally representative data on all Americans, including community dwellers and those living in group quarters such as nursing homes [28]. The response rate for the 2006 ACS was 97.5% [29]. We restricted our sample to respondents in the Public Use Microdata Set who were aged 55 and over who reported that they had ADL limitations as defined by their response to a question asking them if, due to a physical, mental, or emotional condition lasting 6 months or more, they had difficulty doing any of the following activities: “dressing, bathing, or getting around inside the home”. Our sample included older individuals with ADL limitations who reported they were non-Hispanic whites (n = 55,040), or from one of the following ethnic groups: Hawaiian/Pacific Islander (n = 54), Vietnamese (n = 203), Korean (n = 131), Japanese (n = 193), Filipino (n = 309), Asian Indian (n = 169), and Chinese (n = 404). Thus, our total unweighted sample size was 56,503 respondents with ADL limitations who were aged 55 or older. Biracial respondents and those who reported they were from more than one Asian ethnic subgroup were not included in our analyses.

### 2.2. Measures

Institutionalization refers to individuals in nursing homes, in-patient hospice facilities, psychiatric hospitals and adult correctional facilities [30]. Asian ethnicity was divided into eight categories, Non-Hispanic white, Hawaiian/Pacific Islander, Vietnamese, Korean, Japanese, Filipino, Asian Indian and Chinese. Respondents were asked if, due to a physical, mental, or emotional condition lasting 6 months or more, they had difficulty doing any of the following activities: “learning, remembering or concentrating” (classified as cognitive problems). Age was grouped into 10-year age groups: 55–64 (reference category), 65–74, 75–84, 85 or older. Male was the reference category for gender. Education was divided into five categories: Primary School, High School—No Diploma, High School Diploma, Bachelors’ Degree and Graduate Degree (reference category). Citizenship was divided into non-citizens (Reference category) and citizens (either by birth or by naturalization). Language spoken at home was dichotomized into English (Reference category) *versus* other languages. For the first logistic analysis which included all Asian subgroups and Non-Hispanic whites, the latter was chosen as the reference category because it represented the largest group. In the second logistic analysis which excluded Non-Hispanic whites, the Chinese respondents represented the largest Asian ethnic group and were therefore used as the reference category. Information on income was not available for institutionalized older adults. However, in Table 1 we provided information on percentage of non-institutionalized respondents of each ethnicity who were living below or at 100–199%, 200–299%, 300–399%, 400–499% or 500% or higher of the 2006 U.S. federal poverty line. 

### 2.3. Data Analysis

We compared the eight cultural groups on the above listed characteristics. We reported the weighted prevalence and national population estimates. The first logistic regression analysis examined if a difference existed in the age and sex adjusted odds of institutionalization between Non-Hispanic whites and each of the seven Asian ethnicities. Further investigations explored whether these associations were attenuated when the additional sociodemographic characteristics (*i.e.*, marital status, education level), health characteristics (*i.e.*, cognitive problems) and immigration factors (e.g., citizenship, English spoken at home) were included in the analysis. 

A second logistic regression analysis was restricted solely to AAPI older adults with ADL limitations in order: (1) to determine the extent to which the adjusted odds of institutionalization vary among the seven Asian ethnicities; (2) to examine if ethnicity provides any additional explanatory power after characteristics known to influence the likelihood of institutionalization were included in the analysis (e.g., U.S. citizenship status, whether English was spoken at home, cognitive problems, age); and (3) to identify the characteristics associated with institutionalization among Asian elderly with disabilities. For objective 2, the change in the Nagelkerke R Square between a model adjusted for age, sex, marital status, education level, cognitive problems, citizenship, and whether English was spoken at home and a model adjusted for all these factors and ethnicity was calculated.

## 3. Results

The prevalence of institutionalization among older AAPI adults with ADL limitations varied substantially from 4.7% of Asian Indians to 18.8% of Korean Americans (please see Table 1). All AAPI groups had substantially and significantly (*p* < 0.001) lower rates of institutionalization than the non-Hispanic white Americans with ADL limitations (23.8%). The prevalence of cognitive problems among older adults with ADL limitations was much higher in certain groups (e.g., Vietnamese, 73.9%; Japanese, 69.9%; Korean, 69.8%), in comparison to others (e.g., Filipino, 59.8%; non-Hispanic white, 57.6%) (*p* < 0.001). The Vietnamese had the highest rate of cognitive problems, yet one of the lowest rates of institutionalization. The estimated numbers of institutionalized older adults with ADL limitations nationally were below 1,000 in several AAPI groups (*i.e.*, Hawaiian/Pacific Islanders, Vietnamese and Asian Indians). 

The age profile of AAPI older adults with ADL limitations varied by ethnic group. Japanese were disproportionately aged 85 and over (41.2%). In contrast, only 12.4% of Native Hawaiian/Pacific Islanders were aged 85 and over. Approximately 30% of non-Hispanic whites with ADL limitations were 85 and over. 

As shown in Table 2, despite the fact that all respondents in this sample had ADL limitations, Vietnamese, Japanese, Filipino, Chinese and Asian Indians had significantly lower age and sex adjusted odds of institutionalization than non-Hispanic whites. Koreans and Hawaiian Pacific Islanders did not differ significantly from non-Hispanic whites. When full adjustments were made (see Model 2), the findings were similar. The odds of institutionalization were modestly higher among females (OR = 1.08; 95% CI = 1.03, 1.13) and those with only a primary school education (OR = 1.46; 95% CI = 1.30, 1.64) in comparison to those with a graduate degree. Those who spoke English at home had 66% higher odds of institutionalization (OR = 1.66; 95% CI = 1.53, 1.81). The odds of institutionalization were dramatically higher among those with cognitive problems (OR = 2.63; 95% CI = 2.51, 2.76), citizens (OR = 2.20; 95% CI=1.73, 2.80) in comparison to non-citizens, and those 85 and over (OR = 6.25; 95% CI = 5.78, 6.76) in comparison to those aged 55–64, and substantially lower among married respondents (OR = 0.43; 95% CI = 0.41, 0.46). In this analysis which included all the AAPI groups and non-Hispanic whites, all but one result was in keeping with our hypotheses. The one exception was education. We had anticipated that those who were poorly educated would be less likely to learn about nursing home options and therefore less likely to use them. This was not the case in this analysis.

**Table 1:** Description of Study Participants and Weighted National Estimates Grouped by Ethnicity of Individuals Aged 55 and over with Activities of Daily Living. (Source: 2006 American Community Survey).

Weighted estimates according to ethnicity								
Characteristic	Chinese	Pacific Islander	Vietnamese	Korean	Japanese	Filipino	Asian Indian	Non-Hispanic White
	N = 43,218	N=5,966	N = 22,460	N = 14,148	N = 17,258	N = 32,102	N = 18,671	N = 4,915,191
**Institutionalization**								
** Not Institutionalized**	37,500	5,286	21,220	11,489	14,431	29,159	17,799	3,743,339
	(86.8%)	(88.6%)	(94.5%)	(81.2%)	(83.6%)	(90.8%)	(95.3%)	(76.2%)
** Institutionalized**	5,718	680	680	1,240	2,659	2,827	872	1,171,852
	(13.2%)	(11.4%)	(5.5%)	(18.8%)	(16.4%)	(9.2%)	(4.7%)	(23.8%)
**Gender**								
** Male**	16,780	2,764	9,991	4,740	6,256	10,636	6,733	1,712,285
	(38.8%)	(46.3%)	(44.5%)	(33.5%)	(36.2%)	(33.1%)	(36.1%)	(34.8%)
** Female**	26,438	3,202	12,469	9,408	11,002	21,466	11,938	3,202,906
	(61.2%)	(53.7%)	(55.5%)	(66.5%)	(63.8%)	(66.9%)	(63.9%)	(65.2%)
**Age**								
** 55–64**	6,316	1,990	5,167	1,697	1,919	6,400	4,333	1,060,053
	(14.6%)	(33.4%)	(23.0%)	(12.0%)	(11.1%)	(19.9%)	(23.2%)	(21.6%)
** 65–74**	8,346	1,462	6,351	3,251	2,244	6,150	5,416	909,278
	(19.3%)	(24.5%)	(28.3%)	(23.0%)	(13.0%)	(19.2%)	(29.0%)	(18.5%)
** 75–84**	16,081	1,776	7,166	6,132	5,983	11,463	5,470	1,481,325
	(37.2%)	(29.8%)	(31.9%)	(43.3%)	(34.7%)	(35.7%)	(29.3%)	(30.1%)
** 85+**	12,475	738	3,776	3,068	7,112	8,089	3,452	1,464,535
	(28.9%)	(12.4%)	(16.8%)	(21.7%)	(41.2%)	(25.2%)	(18.5%)	(29.8%)
**Marital Status**								
** Not Married**	23,476	3,524	10,774	8,611	9,561	16,598	8,655	3,086,557
	(54.3%)	(59.1%)	(48.0%)	(60.9%)	(55.4%)	(51.7%)	(46.4%)	(62.8%)
** Married**	19,742	2,442	11,686	5,537	7,697	15,504	10,016	1,828,634
	(45.7%)	(40.9%)	(52.0%)	(39.1%)	(44.6%)	(48.3%)	(53.6%)	(37.2%)
**Education (Highest completed)**								
** Primary School**	19,642	2,353	9,404	3,773	2,782	8,834	7,895	854,930
	(45.4%)	(39.4%)	(41.9%)	(26.7%)	(16.1%)	(27.5%)	(42.3%)	(17.4%)
** High School—No Diploma**	4,191	857	4,295	2,076	2,737	3,043	2,201	771,061
	(9.7%)	(14.4%)	(19.1%)	(14.7%)	(15.9%)	(9.5%)	(11.8%)	(15.7%)
** High School Diploma**	13,508	2,580	6,941	5,551	8,639	12,377	4,658	2,661,023
	(31.3%)	(43.2%)	(30.9%)	(39.2%)	(50.1%)	(38.6%)	(24.9%)	(54.1%)
** Bachelors’ Degree**	3,670	76	1,322	1883	2,293	6,557	2,216	390,941
	(8.5%)	(1.3%)	(5.9%)	(13.3%)	(13.3%)	(20.4%)	(11.9%)	(8.0%)
** Graduate Degree**	2,207	100	498	865	807	1,291	1,701	237,236
	(5.1%)	(1.7%)	(2.2%)	(6.1%)	(4.7%)	(4.0%)	(9.1%)	(4.8%)
**Poverty Level ^1^**								
** Under poverty line**	7,498	996	4,910	3,052	1,444	3,511	2,590	610,078
	(20.0%)	(18.8%)	(23.1%)	(26.6%)	(10.0%)	(12.0%)	(14.6%)	(16.3%)
** 100–199%**	10,218	975	4,465	3,700	2,539	6,236	2,984	1,057,774
	(27.2%)	(18.4%)	(21.0%)	(32.2%)	(17.6%)	(21.4%)	(16.8%)	(28.3%)
** 200–299%**	4,993	1,298	4,343	1,319	2,301	2,944	2,606	713,717
	(13.3%)	(24.6%)	(20.5%)	(11.5%)	(15.9%)	(10.1%)	(14.6%)	(19.1%)
** 300–399%**	4,410	291	2,210	637	2,491	4,882	2,976	462,692
	(11.8%)	(5.5%)	(10.4%)	(5.5%)	(17.3%)	(16.7%)	(16.7%)	(12.4%)
** 400–499%**	2,870	623	1,762	479	904	3,559	2,210	297,961
	(7.7%)	(11.8%)	(8.3%)	(4.2%)	(6.3%)	(12.2%)	(12.4%)	(8.0%)
** 500%+**	7,511	1,103	3,530	2,302	4,752	8,027	4,433	601,117
	(20.0%)	(20.9%)	(16.6%)	(20.0%)	(32.9%)	(27.5%)	(24.9%)	(16.1%)
** Cognitive Problems**								
** No Problems**	14,440	2,196	5,854	4,272	5,192	12,894	7,095	2,086,130
	(33.4%)	(36.8%)	(26.1%)	(30.2%)	(30.1%)	(40.2%)	(38.0%)	(42.4%)
** Some Cognitive Problems**	28,778	3,770	16,606	9,876	12,066	19,208	11,576	2,829,061
	(66.6%)	(63.2%)	(73.9%)	(69.8%)	(69.9%)	(59.8%)	(62.0%)	(57.6%)
**Citizenship**								
** American by birth or naturalization**	35,177	5,023	16,954	10,272	16,428	26,096	10,455	4,856,583
	(81.4%)	(84.2%)	(75.5%)	(72.6%)	(95.2%)	(81.3%)	(56.0%)	(98.8%)
** Not a citizen**	8,041	943	5,506	3,876	830	6,006	8,216	58,608
	(18.6%)	(15.8%)	(24.5%)	(27.4%)	(4.8%)	(18.7%)	(44.0%)	(1.2%)
**Language spoken at home**								
** Speaks English at home**	3,409	2,629	2,346	556	9,212	3,464	3,535	4,519,002
	(7.9%)	(44.1%)	(10.4%)	(3.9%)	(53.4%)	(10.8%)	(18.9%)	(91.9%)
** Doesn’t Speak English at home**	39,809	3,337	20,114	13,592	8,046	28,638	15,136	396,189
	(92.1%)	(55.9%)	(89.6%)	(96.1%)	(46.6%)	(89.2%)	(81.1%)	(8.1%)

All *p*-values <0.001 These weighted national estimates were based on an unweighted sample size of 56,503 respondents to the 2006 ACS who were aged 55 or older and who had ADL limitations. The unweighted sample size for each ethnic group was as follows: Chinese (n = 404), Hawaiian/Pacific Islander (n = 54), Vietnamese (n = 203), Korean (n = 131), Japanese (n = 193), Filipino (n = 309), Asian Indian (n = 169) and non-Hispanic whites (n = 55,040). ^1^ Poverty level information based on the 2006 U.S. federal poverty line was only available for non-institutionalized respondents.

**Table 2:** Logistic Regression Analysis of Institutionalization among Individuals Aged 55 and over with Limitations in Activities of Daily Living. (Includes Non-Hispanic Whites and 7 AAPI Ethnicities) Source: 2006 American Community Survey.

Characteristic	Model 1	Model 2
	OR (95% Confidence Interval)	OR (95% Confidence Interval)
**Race**		
** Non-Hispanic White**	1.00 (Ref)	1.00 (Ref)
** Chinese **	0.44 (0.34, 0.58)	0.70 (0.53, 0.93)
** Hawaiian/ Pacific Islander **	0.55 (0.25, 1.19)	0.55 (0.25, 1.21)
** Vietnamese **	0.22 (0.12, 0.37)	0.31 (0.18, 0.55)
** Korean **	0.72 (0.48, 1.09)	1.25 (0.82, 1.92)
** Japanese**	0.50 (0.34, 0.73)	0.58 (0.39, 0.87)
** Filipino**	0.31 (0.21, 0.45)	0.51 (0.35, 0.75)
** Asian Indian**	0.17 (0.09, 0.33)	0.29 (0.15, 0.57)
**Gender**		
** Male**	1.00 (Ref)	1.00 (Ref)
** Female**	1.33 (1.27, 1.39)	1.08 (1.03, 1.13)
**Age **		
** 55–64**	1.00 (Ref)	1.00 (Ref)
** 65–74**	2.19 (2.00, 2.39)	2.31 (2.11, 2.53)
** 75–84**	4.34 (4.01, 4.69)	4.01 (3.71, 4.35)
** 85+**	8.13 (7.53, 8.77)	6.25 (5.78, 6.76)
**Marital Status**		
** Not married**	---	1.00 (Ref)
** Married**	---	0.43 (0.41, 0.46)
**Education (Highest completed)**		
** Graduate Degree**	---	1.00 (Ref)
** Bachelors’ Degree**	---	1.24 (1.09, 1.42)
** High School Diploma**	---	1.36 (1.22, 1.53)
** High School—No Diploma**	---	1.32 (1.17, 1.49)
** Primary School**	---	1.46 (1.30, 1.64)
**Cognitive Problems**		
** No Problems**	---	1.00 (Ref)
** Some Cognitive Problems**	---	2.63 (2.51, 2.76)
**Citizenship**		
** Not a citizen**	---	1.00 (Ref)
** American by birth or naturalization**	---	2.20 (1.73, 2.80)
**Language spoken at home**		
** Doesn’t Speak English at home**	---	1.00 (Ref)
** Speaks English at home**	---	1.66 (1.53, 1.81)

When non-Hispanic whites were excluded from the analysis (See Table 3), there was considerable variation among the seven Asian ethnicities: in comparison to Chinese Americans with ADL limitations, the fully adjusted odds of institutionalization were 63% lower for Vietnamese (OR = 0.37; 95% CI = 0.19, 0.74) and 69% lower for Asian Indians (OR = 0.31; 95% CI = 0.14, 0.71). The other ethnicities did not differ significantly from Chinese respondents. When all the other factors were in the logistic regression analysis of institutionalization, the addition of ethnicity to the model added an additional 3.2% explanatory value (*p* < 0.001; analysis not shown). 

**Table 3:** Logistic Regression Analysis of Institutionalization among Individuals Aged 55 and over with Limitations in Activities of Daily Living. (Includes 7 AAPI Ethnicities Only, Non-Hispanic Whites Excluded) Source: 2006 American Community Survey.

Characteristic	OR (95% Confidence Interval)
**Chinese **	1.00 (Ref)
**Hawaiian/Pacific Islander **	0.69 (0.27, 1.80)
**Vietnamese **	0.37 (0.19, 0.74)
**Korean**	1.62 (0.93, 2.81)
**Japanese**	0.65 (0.36, 1.17)
**Filipino**	0.71 (0.43, 1.19)
**Asian Indian**	0.31 (0.14, 0.71)
**Male**	1.00 (Ref)
**Female**	0.83 (0.56, 1.24)
**55–64**	1.00 (Ref)
**65–74**	1.81 (0.79, 4.14)
**75–84**	2.70 (1.27, 5.74)
**85+**	5.18 (2.43, 11.06)
**Married**	1.00 (Ref)
**Not married**	1.72 (1.13, 2.60)
**Graduate Degree**	1.00 (Ref)
**Bachelors’ Degree**	1.00 (0.35, 2.86)
**High School Diploma**	1.30 (0.51, 3.32)
**High School—No Diploma**	2.11 (0.80, 5.60)
**Primary School**	0.87 (0.34, 2.25)
**No Cognitive Problems**	1.00 (Ref)
**Some Cognitive Problems**	3.80 (2.30, 6.28)
**Not a citizen**	1.00 (Ref)
**American by birth or naturalization**	1.07 (0.67, 1.73)
**Doesn’t Speak English at home**	1.00 (Ref)
**Speaks English at home**	2.26 (1.42, 3.61)

Model 1: Variables included = gender, age, marital status, education level, cognitive problems, US citizenship, Speaks English at home; Model 1 Chi-Square 117.6; *p* < 0.001; Nagelkerke R-square = 15.4%; Final Model: Variables included = Model 1 variables and ethnic group; Final Model Chi-Square 142.5; *p* < 0.001; Nagelkerke R-square = 18.6%; Change in Model Chi-Square due to addition of ethnic group: 24.9; *p* < 0.001; Change in Nagelkerke R-square due to addition of ethnic group: 3.2%.

Among Asian elderly with ADL limitations, institutionalization is associated with older age, cognitive problems, unmarried status and speaking English at home. In particular, those 75–84 had 2.7 times higher odds (95% CI = 1.27, 5.74) and those 85 and over had five times higher odds (OR = 5.18; 95% CI = 2.43, 11.06) of institutionalization than those aged 55–64. Those with cognitive problems had approximately four times higher odds of institutionalization (OR = 3.80; 95% CI = 2.30, 6.28). The unmarried had 72% higher odds (OR = 1.72; 95% CI = 1.13, 2.60) of institutionalization compared to their married peers. Those who spoke English at home had more than twice the odds (OR = 2.26; 95% CI = 1.42, 3.61) of institutionalization in comparison to their peers who did not speak English at home. All of these factors were in keeping with our original hypotheses. However, two additional factors (*i.e.*, American citizenship and education level) did not reach statistical significance. 

## 4. Discussion and Conclusions

This study provides, to our knowledge, the first analysis of factors associated with institutionalization in seven AAPI subgroups in a nationally representative sample of individuals aged 55 and over with ADL limitations. There are two key findings: First, AAPI groups vary markedly in the prevalence of institutionalization among their disabled older adults, underlining the importance of considering each ethnicity separately rather than grouping AAPI ethnicities together. Second, five of the seven AAPI ethnicities had lower rates of institutionalization than non-Hispanic whites, even after adjustments for most of the key risk factors for institutionalization in the general population (e.g., cognitive problems, age, marital status) and factors thought to facilitate or impede institutionalization among Asian Americans (*i.e.*, citizenship, acculturation as measured by English language use at home). 

There were striking differences in the prevalence rates of institutionalization of disabled elderly among the AAPI subgroups (ranging from 4.7% in Asian Indians to 18.8% among Koreans). Some of this variability may be due to difference in each ethnicity’s age structure of the 55+ population and prevalence of cognitive impairments. However, even after adjusting age, sex, cognitive problems and many other possible covariates, older adults with ADL limitations who were Asian Indian and Vietnamese had significantly lower odds of institutionalization than Chinese Americans. These variations may be a function of immigration history, availability of adult children to provide personal care assistance, and cultural acceptability of institutionalization. 

These variations underline the importance of public health professionals paying closer attention to ethnic variations within Asian populations in order to better tailor service delivery. Health care providers need to be aware that ethnic traditions and filial piety play a significant role in these families [31,32,33,34]. 

Non-Hispanic whites had significantly higher odds of institutionalization when compared to five of the seven AAPI subgroups and these differences could not be explained by citizenship or acculturation as measured by English language use at home. Although each of the Asian subgroups has its own immigration history, language and culture, the existing body of literature appears to indicate that all AAPI cultures are more collectivist than individualist. In other words, in these cultures, the goals of the group are believed to be more important than those of the individuals [35,36,37,38]. Family-oriented values result in lower willingness of AAPI adult children to place an elderly parent in a nursing home [38]. 

Asian Americans who are less acculturated tend to uphold traditional values and perceive institutionalization as stigmatizing [8,11,12]. Among AAPIs, speaking English at home (which we anticipated was a marker of acculturation) was associated with twice the odds of institutionalization. 

With almost one-half of national spending on nursing home care coming from Medicaid payments [39], we had assumed citizenship would play a major role in mediating the link between ethnicity and institutionalization in the AAPI population because only citizens are eligible for Medicaid Nursing Home supplements. In the AAPI population, there was no significant association between institutionalization and citizenship. 

We had anticipated that those who were poorly educated would be less likely to learn about nursing home options and therefore less likely to use them. This was not the case in this analysis. Consistent with previous research, this study found that the odds of institutionalization were higher for those who had cognitive problems and those who were older [40]. 

Although the national numbers of disabled elderly in many AAPI subgroups are very low, the AAPI elderly population is projected to grow by 250% between 2000 and 2025 [41]. It is the opinion of the authors that it is essential that disabled AAPI older adults have improved access to a continuum of care that is linguistically appropriate and culturally sensitive. Drawing heavily upon recent Australian and Canadian work in the area, the following paragraphs highlight potential policy recommendations to address the needs of AAPI older adults in residential care.

There are three options for the residential care of older AAPIs: Ethno-specific nursing homes, mainstream nursing homes which contain language-specific clusters organized by floor or wing and mainstream nursing homes [42]. The first two options are designed to be culturally responsive to the needs of non-English speaking residents. A position paper of the Ethnic Communities’ Council of Victoria (Australia) suggests that ethno-specific nursing homes are relatively rare and typically have long waitlists [42]. High demand and long-wait lists for ethno-specific nursing homes have also been noted in Canada [43,44] and in the United States [45]. The second option, culturally distinct floors within mainstream nursing homes, provides an interesting option within already established institutions. Most existing dementia-specific care units in mainstream nursing homes are of relatively small size. Therefore nursing homes which wish to provide ethno-specific units have the opportunity to reorganize their staffing and clients by language skills to convert one or their dementia-specific special care units to language specific units for those with dementia from particular language groups [42,44,45].

Policy suggestions for mainstream residential care facilities which serve some AAPI patients include offering these individuals at least one menu choice at each meal which is culturally appropriate, hiring at least one bilingual social worker and some bilingual nursing staff, as well as providing easy access to interpreters [42,44,45]. Training workshops sensitizing staff to cultural issues can improve services to potentially isolated and lonely non-English speakers. Links between culturally specific community groups such as Korean churches or Japanese temples and nursing homes can provide improved access of non-English speaking AAPI residents to linguistically similar visitors [34,44].

This study has several limitations which must be considered. It is possible that the variation in institutionalization rates may result from Asian Americans having more children than non-Hispanic whites and therefore having more adult children with whom to coreside if disability strikes. However, previous research suggests that the differences in number of adult children are unlikely to be a major contributor to the white-Asian differences in institutionalization [7].

Structural differences such as limited economic resources could also contribute to lower institutionalization rates among AAPI elders in comparisons to non-Hispanic whites. Unfortunately, we did not have information on the institutionalized respondents’ income so we could not include income in our logistic regression analyses. However, we did have information on non-institutionalized older adults’ income levels (see Table 1) as measures by their level with respect to the 2006 U.S. federal poverty line. These data do not suggest income plays a major role: Non-Hispanic whites had higher institutionalization rates than all AAPI groups whether the Asian ethnicities have a greater proportion with high income than non-Hispanic whites (e.g., Japanese, Asian Indians) or whether they had a greater proportion of older adults living under the 2006 U.S. federal poverty line than non-Hispanic whites (e.g., Vietnamese).

Another substantial limitation of this study is the fact that the public use microdata file of the ACS 2006 aggregated those in “nursing homes, in-patient hospice facilities, psychiatric hospitals and adult correction facilities” into the same “institutionalized” category. We have focused our discussion on long-term care use because we anticipate that the latter three forms of institutionalization are unlikely to include substantial numbers of older AAPIs with ADL limitations. In 2007, there were 2.4 million Asian Americans aged 55 or older [46]. Yet in all State and Federal prisons there were only 3,000 prisoners aged 55 and over who defined themselves as American Indians, Alaska Natives, Asians, Native Hawaiians, or other Pacific Islanders [47]. With respect to institutional hospice care, more than 90% of hospice days are provided in patients’ home, not institutions, and of the small minority who receive hospice services in institutions, only 10% were neither non-Hispanic white nor Black [48]. In 2000, there were approximately 80 inpatient psychiatric treatment beds per 100,000 people of all ages and races in the U.S. population [49]. Asian Americans have a lower institutionalization rate in psychiatric hospitals than non-Hispanic whites, Blacks, and American Indians [50]. 

Despite these limitations, this is the first study to provide representative population-based data of community-dwelling and institutionalized older adults with ADL limitations from seven AAPI ethnic groups. Five of the seven AAPI groups had significantly lower odds of institutionalization than disabled non-Hispanic whites, even after adjustment for most of the known risk factors for institutionalization. When the sample was restricted to AAPI respondents, the addition of ethnicity to the logistic regression model added an additional 3.2% explanatory value. This indicates that individual ethnic groups are a relatively robust predictor of institutionalization over and above citizenship status, whether English is spoken at home, age, gender and cognitive problems. The findings of this study provide important empirical data to help public health professionals working with AAPI older adults with ADL limitations. 
